# Commentary: “Physical time within human time” and “Bridging the neuroscience and physics of time”

**DOI:** 10.3389/fpsyg.2023.1063327

**Published:** 2023-09-28

**Authors:** Simon Grondin

**Affiliations:** École de Psychologie, Université Laval, Québec, QC, Canada

**Keywords:** time perception, psychophysics, psychological time, human times, Weber's law

Gruber et al. (2022) propose an interesting way of addressing the difficult, but fundamental problem of the nature of time. This problem is a long lasting one for physicists, mathematicians and psychologists or neuroscientists (see for example Buccheri et al., 2003; Buonomano and Rovelli, 2022). Gruber and collaborators propose an adaptation of the Information Gathering and Utilizing System (IGUS), acknowledging that both illusory and veridical times exist and guide behavior. This commentary will focus more on the part of the article of Gruber and collaborators devoted to the findings/concepts extracted from the literature in experimental psychology, which is more closely linked to the *global* (or even *local*) *presentism* position than to the *static eternalism* position that Buonomano and Rovelli describe.

## 1. About perception and psychophysics in general

A first impression that comes during the reading of the article of Gruber et al. is that fundamentally, what could be presented as opposing times, one inside and one outside the cranium, extends beyond the question of human time. Ultimately, a fundamental question that we have to face could be posited as follows: is there anything like a physical reality, and if there is, is it possible to capture it? Posited another way, and assuming there is such an objective world outside of us, one can ask to what extent we are prisoners of our sensory organs, limited in our representations of outer world and, consequently, in our representation of what time is or could be.

It is for capturing the links between material world and mental world that *psychophysics* was founded (Fechner, 1860). This field of research has provided methods to quantify the links between the physical world and the sensation we derive from it and which guides our representation of it. Fechner was interested in the relation between sensations and stimulus intensities (outer psychophysics) and the relation between sensations and brain activity (inner psychophysics).^1^ Outer psychophysics provides information mainly about the minimal energy needed to detect a stimulus, about the capability to discriminate stimuli, and about scaling, i.e., the assessment of the psychological value of stimuli as a function of their magnitude. The empirical work in psychophysics led to laws about the relationship between psychological (subjective/mental) world and physical (objective/material) world.

The question now is: can we use psychophysical information (methods and laws), developed to quantify and understand sensations, for addressing the problem of human time? Answering this question requires first to acknowledge some difficulties. Within such a perspective, what is the status of time? Should time be treated like a dimension or like a sense? If it's treated as a dimension, what is it the dimension of? Is it “the” fourth dimension (the block universe), or simply one dimension among several others? If time is treated like a sense, maybe psychophysics could help (Eisler, 1976; Glicksohn and Hadad, 2012; Kornbrot et al., 2013). However, for studying time in the light of psychophysics, we have first to acknowledge that, strictly speaking, although we can define time intervals with sensory signals, there is no “time stimuli” *per se*. As well, there is no “time receptors,” like we have, for instance, the retina or the cochlea for studying properties belonging to vision and audition, respectively. Along the same line, there is apparently no such a thing like a “time cortex,” a part of the brain dedicated to the processing of temporal information as is the case with the visual or auditory cortex for processing visual or auditory information, respectively. The brain, as a whole, can be seen as an inherently temporal organ (Buonomano and Rovelli, 2022), but when time comes to find a temporal resolution, there is a need for a contribution from several cortical and subcortical structures (Grondin, 2010).

As is the case for the perception of sensory stimuli, time perception will sometimes result from a contribution of top-down processes (a taking-into-account process, to use Helmholtz terminology), and the specific parameters (organization in time) of sensory stimuli marking time intervals will sometimes lead to illusions, i.e., an impression that something is there (occurred at a given moment, in the case of time; see for example ten Hoopen et al., 2008; for the description of the time-*shrinking illusion*) when it is not. Illusions are indeed neither a mirage, nor a hallucination; it simply shows the normal functioning of the brain.

## 2. About the continuity of psychological time

In their article, Gruber and collaborators take the direction of the flow of psychological time and review different notions and findings related to persistence, change/motion, temporal order, and specious present. The general question addressed is whether the flow of psychological time is punctuated by any interruption or discontinuity (see Chapter 3 in Grondin, 2020). From the very start, adaptation requires distinguishing successiveness from simultaneity; hearing, for example, is a clear case illustrating the need to efficiently integrate and segregate elements of information occurring in the flow of time (Bregman, 1989).

Gruber et al. brought to our attention numerous relevant examples to illustrate that there is a gap between physical reality and conscious perception. There are indeed several types of “temporal displacements” (Vicario, 2003, 2005). One fascinating example is that of the *flash-lag effect*. This phenomenon could be demonstrated when a flashing object and a moving target should be aligned. The flashed object will be perceived to lag behind the position of moving target (Hubbard, 2014). Note that this flash-lag effect could be viewed as a special case of another phenomenon called *representational momentum*. This phenomenon refers to the displacement of a moving target further along its anticipated path of motion (Hubbard, 2005). This phenomenon could be viewed as a mechanism compensating for delays in awareness due to neural processing latencies.^2^

Gruber et al. make some room to the notion of specious present in the “two time” debate. The idea here is to acknowledge that there must be some continuity within a given time window to assure that there is some unity in the flow of information reaching the brain. There is some ambiguity though about the duration of this window, with values reported in the article being 0.5, or 3 s, or even 7 s. One way of addressing this issue is proposed below, based on a classical psychophysical law.

According to Weber's law, the minimum difference between two stimuli (the discrimination threshold) needed to discriminate them depends on the magnitude of the stimuli. More specifically, this difference increases proportionally with the magnitude. In other words, the threshold to magnitude ratio should be constant (the Weber fraction is constant). For the study of human time, one can look at this Weber fraction as a function of physical time (or, to be more careful given the uncertainty about what physical time is, as a function of chronometric time). It turns out that there are instances where the fraction is not constant; in other words, Weber's law doesn't hold (Grondin, 2001). An increase of the Weber fraction for low magnitudes of chronometric time could by easily accounted for mathematically with a generalized version of Weber's law. However, the fact that this fraction increases when intervals to be discriminated are longer than circa 1.2 s (Grondin, 2012), or around 1.5 s, according to Gibbon et al. (1997), is more difficult to explain. Even counting at a 1.6 s pace, in comparison with a 0.8 s pace, will lead to much more variability (Grondin et al., 2015). This disruption in the capability to process a temporal extent could be interpreted as a limitation in the flow of psychological time; it could be viewed as a tool to quantify the “specious present” Gruber et al. referred to, and may also reflect a fundamental temporal limitation of short-term memory. And by the way, it turns out that humans have a way to go round this limitation by segmenting a time span into smaller chunks by using, for example, an explicit counting strategy (Grondin et al., 1999).

## 3. Concluding remarks

What is human time? Just unifying psychological times is a challenge. There is no human time, but human times: temporal orientation, temporal perspective, temporal order of past events, distance of events in the past, speed of the passage of time, flow of speech or music, tenses in language, to name a few. Buonomano and Rovelli proposed their own taxonomy of time features, acknowledging the need to present time as a multilayered concept. Even within a simple experimental psychology perspective, where we want to keep explanations simple, the questions of the continuity of time and of the sources of time-adapted behavior are blurred by a multiplicity of findings.

Is there a physical/material world outside of us? There could be something, and there could be nothing. Both avenues are unbearable. Consciousness is a cruel coquetry of human existence, but also its most fascinating charm. Time is arguably at the heart of consciousness, considering the need that the brain constantly rearranges the timing of events, as Gruber, Block and Montemayor noted in their target article. Addressing the problem posed by the idea/notion of time and exploring the content of human time, as Gruber, Block and Montemayor have done, and trying to build bridges between physics and neuroscience, as Buonomano and Rovelli propose, is probably a good way to take significant steps toward an understanding of this elusive phenomenon that is that of consciousness.

## Author contributions

The author confirms being the sole contributor of this work and has approved it for publication.

## Footnotes

^1^For additional information about fundamental questions in psychophysics and about the future of psychophysics, please see Hubbard (2020, 2021).

^2^See Grondin and Hubbard (2024) for a review of different types of brief gaps in awareness of the external environment, and of potential disadvantages of awareness in some perceptual and cognitive processes.
